# Clinicopathological Significance of Vimentin and Cytokeratin Protein in the Genesis of Squamous Cell Carcinoma of Cervix

**DOI:** 10.1155/2016/8790120

**Published:** 2016-04-14

**Authors:** Nazik Elmalaika O. S. Husain, Ali Yousif Babiker, Aqel S. Albutti, Mohammed A. Alsahli, Salah M. Aly, Arshad H. Rahmani

**Affiliations:** ^1^Department of Pathology, College of Medicine, Omdurman Islamic University, 382 Omdurman, Sudan; ^2^Department of Medical Laboratories, College of Applied Medical Sciences, Qassim University, Buraidah 6666, Saudi Arabia; ^3^Department of Histopathology and Cytology, College of Medical Laboratories Science, University of Sciences and Technology, Omdurman, Sudan; ^4^College of Veterinary Medicine, Suez Canal University, Ismailia, Egypt

## Abstract

Cervical cancer is one of the commonest types of cancers worldwide especially in developing countries. Intermediate filaments protein family has shown a role in the diagnosis of various cancers, but a few studies are available about the vimentin and cytokeratin roles in the cervical cancer. This case control study aimed to interpret the expression of vimentin and cytokeratin proteins in the development and progression of cervical cancer and its correlation with clinicopathological features. The cytoplasmic expression of vimentin was observed in 40% of cases, but not in inflammatory lesions of cervix. It was noticed that vimentin expression was increasing significantly with high grade of the tumour. Cytokeratin expression was observed in 48.33% and it was noticed that the expression was 62.5% in well differentiated (G1), 45% in moderately differentiated (G2), and 41.66% in poorly differentiated carcinoma, yet statistically insignificant. The expression of vimentin and cytokeratin proteins was not significantly associated with age groups. The current findings concluded a possible role of vimentin in the development and progression of cervical cancer and vimentin marker will be useful in the diagnosis and grading of cervical cancer.

## 1. Introduction

Cervical cancer is the second commonest cancer among females worldwide [1]. The incidence of uterine cervix cancer is increasing worldwide and there are various factors that act as culprit in the development and progression of cervical cancer. The exact reason of development and progression of cancer including cervical cancer is not fully explained. Most cases occur in developing world where effective screening systems are not available [2]. As per research findings, it is confirmed that various factors show role in cancer development and progression including structural and function alteration in various genes, smoking, chewing, and Human Papillomavirus (HPV) infection [3, 4]. Various markers are in practice to diagnose cervical cancer but still a few studies were made on intermediate filament protein family markers role in the cervical cancer.

Intermediate filament (IF) protein family such as cytokeratin and vimentin has been suggested to play a role in the diagnosis of cervical cancer. Cytokeratin belongs to the IF protein family and is categorised into type I with CK9–CK23 and type II that constitutes CK1–CK8 subclasses [5]. Vimentin is one of the other important IF proteins of the mesenchymal cells and is the key motif of the cytoskeleton. Increased expression of cytokeratin and vimentin is associated with development and progression of cancers. Earlier study has confirmed the expression of vimentin and cytokeratin in breast cancer [6, 7] and vimentin positivity has been commonly noticed in numerous types of cancer and was correlated with a sign of an epithelial-mesenchymal transition [8–12]. This study aims to interpret the expression of the vimentin and cytokeratin protein in the development and progression of cervical cancer and make relationship between vimentin and cytokeratin protein expression based on clinicopathological characteristics.

## 2. Materials and Methods

This was a case control study which was done on Sudanese patients diagnosed histopathologically with cervical cancer in the Histopathology Department at the National Health Laboratory (or in different histopathology laboratories) in Khartoum State, Sudan.

**Figure 1:**
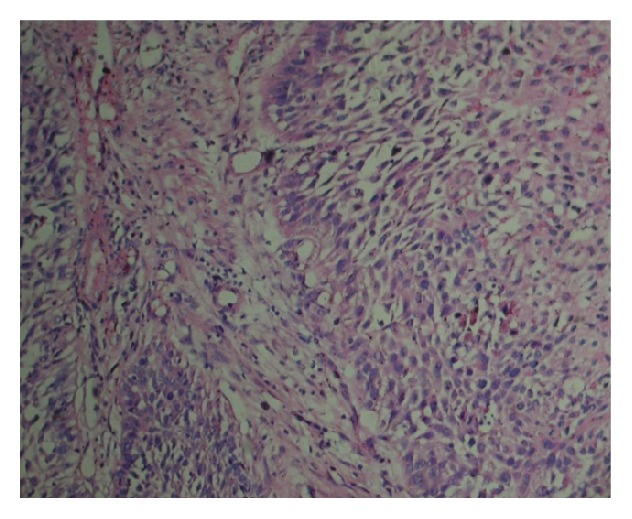
H&E staining showing squamous cell carcinoma. Org. Mag. ×400.

A total of sixty patients of cervix cancer cases and 10 cases of benign condition of cervix were collected for evaluation of the expression of vimentin and cytokeratin in development and progression of cervix cancer. The cases were categorised on the basis of grade into grade I (*n* = 16), grade II (*n* = 20), and grade III (*n* = 24). The sections were cut with 4 *μ*m and histopathological changes/differentiations were evaluated via H&E staining (Figure 1).

### 2.1. Immunohistochemistry

Sections of 5 *μ*m thicknesses were cut and mounted onto polylysine-coated slides that were stained for vimentin and cytokeratin via immunohistochemistry staining methods described earlier [13]. Briefly, after the tissue sections were deparaffinized by the treatment of xylene and after hydration, slides were kept for antigen retrieval in microwave for 20–25 mints, in citrate buffer with pH (6.0), followed by washing with PBS buffer three times each for 3 minutes. Endogenous peroxidase activity was blocked through 0.3% hydrogen peroxide block; then slides were incubated for blocking according to manufactures instruction and then three changes of PBS (pH 7.0) buffers were given. Sections were incubated overnight at 4°C with mouse monoclonal anti-human ck and vimentin antibody. Thereafter, slides were incubated for 40 min at room temperature with biotinylated rabbit anti-mouse antibody, followed by incubation with streptavidin-biotin enzyme complex for 30 minutes. Diaminobenzidine (DAB) chromogen was used and then sections were counterstained with hematoxylin. Appropriate negative controls (omission of the primary monoclonal antibody) and positive controls were used throughout staining procedures.

### 2.2. Scoring

Five fields from each section of the tissue were selected and cytoplasmic positive cells were counted. The evaluation of immunopositivity was made taking into account the percentage positivity of tumour cells. The cells were scored as negative or positive and the percentage of positive tumour cells were recorded, which ranged from 0 to 100% [14]. The percentage positivity was graded from 1+ to 3+ as follows: 5–25% as 1+, 25–75% as 2+, and more than 75% as 3+ [14].

### 2.3. Statistical Analysis

Vimentin and cytokeratin expression and its relations based on clinic-pathological characteristics such age, grade, and keratinisation were assessed via Chi square (*λ*)^2^. A *P* value <0.05 was taken as statistically significant.

## 3. Results

All tumours cases were divided based on differentiation and it was well differentiated (G1) in 16 (26.66%), moderately differentiated (G2) in 20 (33.33%), and poorly differentiated (G3) in 24 (40%) patients. Thirty-six (60%) of the nonkeratinized cases were found in the older age groups (≥55) while 24 (40%) were found in the younger age groups (<55 years). Sixty histopathologically confirmed cases of squamous cell carcinoma were included in the study.

### 3.1. Evaluation of Vimentin Expression and Its Correlation with Grade and Age of the Patients

**Figure 2:**
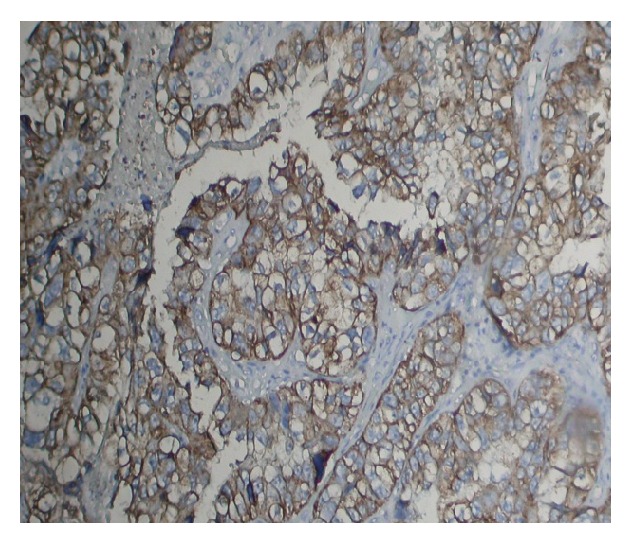
Vimentin expression in the cytoplasm of squamous cell carcinoma (Org. Mag. ×400).

The cytoplasmic expression of vimentin was noticed in 24 (40%) cases (Figure 2) and vimentin expression was not observed in inflammatory lesions of cervix. The expression profile of vimentin was further divided based on differentiation of tumour as 5 (31.25%) into well differentiated (G1), 8 (40%) in moderately differentiated (G2), and 11 (45.83) in poorly differentiated squamous cell carcinoma (Table 1). The differences of expression pattern among different grades were statistically significant and expression was high in poorly differentiated carcinoma. The expression pattern of vimentin was 38% in age group (≥55) and it was 41% in younger group (<55 yeas) and these differences were statistically insignificant (Table 1). Expressional profile was categorised based on keratinisation groups' cases and results revealed that the expression was statistically insignificant in keratinised and nonkeratinised group.

### 3.2. Immunohistochemical Evaluation of Cytokeratin Protein Expression

**Figure 3:**
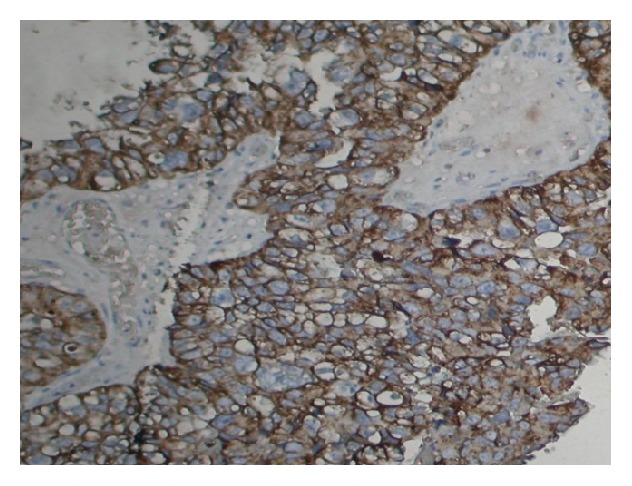
Cytokeratin (CK) expression in the cytoplasm of squamous cell carcinoma (Org. Mag. ×400).

Cytokeratin expression was observed in cytoplasm in 29 (48.33%) of uterine cervix cases (Figure 3). Expression pattern was further correlated with grade of the tumour and it was noticed that CK expression was observed to be well differentiated (G1) in 10 (62.5) patients, moderately differentiated (G2) in 9 (45%) patients, and poorly differentiated in 10 (41.66%) patients. The expression profile of cytokeratin was different in different grades and it was not increased or decreased according to grade and this difference was statistically not significant. The expression pattern was evaluated based on age group and it was noticed that difference in expression among different age groups such as age ≥55 and age less than 55 years was statistically insignificant (Table 1).

The expression pattern of both markers was inversely correlated. Vimentin positivity was increasing according to increased grade of the tumour and CK positivity; though statistically insignificant, it was decreasing among increased grades of the tumour (Table 1).

## 4. Discussion

Uterine cervix cancer is one of the commonest malignancies worldwide including Sudan. Exact reason of development and progression of cervix carcinomas is not well known. But various factors such as alteration in genes function due to human papilloma viruses, smoking, and chewing show role in the cancer development. It is thought that the fact that effective screening systems are not available in Sudan is one of the reasons of high incidence of cancer.

Cervical cancer incidence is higher in older age group populations worldwide and our finding also showed that most cases were in older age group (≥55 age groups). The exact causes of high incidence of cervical cancer in older people are not known properly but it could be due to the collective effects of long time exposures to various carcinogens. Various markers are in use for diagnosis of the cervical cancer but few studies based on intermediate filament markers in cervical cancer are available.

Vimentin is an intermediate filament found predominantly in mesenchymal cells. The vimentin antibody recognizes a 57 kDa IF and labels several types of mesenchymal cells [15]. Altered/high expression of vimentin has been noticed in various types of cancer. Our findings showed that expression of vimentin was 40% and expression pattern was increased according to grade of cervical cancer cases and these differences were statistically significant. The exact reason of the difference in expression pattern of vimentin protein among the different grades of the carcinomas is not fully understood. Previous finding showed that vimentin expression promotes cell migration and invasion in tumour cells of malignancies starting from the breast [16, 17] and other studies reported that upregulation of vimentin expression in various epithelial tumours is intimately associated with the tumour migration, invasion, and metastasis [18]. Abnormal vimentin expression is associated with a high tumour grade such as cancer of the lung [19] and bladder [20] and other studies revealed a positive correlation between extensive vimentin expression and the occurrence of metastasis [21].

A finding based on breast cancer revealed that vimentin expression was noticed in 18% of cases and its expression correlated with high tumour grade and high growth fraction [22] and study results revealed the association between vimentin expression and metastatic progression [23]. Interestingly, a study based on cervical squamous cell carcinoma showed that upregulation of vimentin was inversely related to histologic differentiation, metastasis, and recurrence [24].

In the current study, expression of vimentin was statistically insignificant in relation to age group. However, study based on breast cancer showed that vimentin-positive cancers were more frequently found in younger women [25] and other earlier findings confirmed correlation between basal markers expression and younger patient age [26, 27].

In this study, cytokeratin expression was observed in 29 (48.33%) of uterine cervix cases. Earlier studies have noticed cytokeratin expression in different types of cancer, while, in this study, expression pattern among different grades of cancer was statistically insignificant. An earlier study based on oral squamous cell carcinoma (OSCC) revealed that CK17 and CK13 were detected in 96.2% and 2.9% and CK17 was significantly expressed in well differentiated compared to moderately/poorly differentiated OSCC [28].

## 5. Conclusion

The current finding suggested a possible role of vimentin in the development and progression of cervical cancer and vimentin marker will be helpful in the diagnosis and grading of cervical cancer. Very scanty data are available based on role of both markers in cervix cancer development and progression. Detailed and more studies are needed to assess vimentin and cytokeratin expression in cervical cancer and its correlation with clinical outcome of the patients.

## Figures and Tables

**Table 1:** Expression pattern of cytokeratin and vimentin in squamous cell carcinoma cases.

Clinical parameters	Expression of CK			Expression of vimentin		
	Total cases	% positivity	*P* value	Positive cases	% positivity	*P* value
Grades						
Well differentiated (G1) (*n* = 16)	10	62.5	>0.05	5	31.25	<0.05
Moderately differentiated (G1) (*n* = 20)	9	45		8	40	
Poorly differentiated (G1) (*n* = 24)	10	41.66		11	45.83	
Total cases	29	48.33		24	40	
Age						
<55 years	16	44.44	>0.05	14	38	>0.05
≥55 years	13	54.16		10	41%	
